# Metabolite profiling for biomarkers in *Schistosoma haematobium* infection and associated bladder pathologies

**DOI:** 10.1371/journal.pntd.0006452

**Published:** 2018-04-30

**Authors:** Adewale S. Adebayo, Swapnil D. Mundhe, Henrietta O. Awobode, Olugbenga S. Onile, Atinuke M. Agunloye, Raphael D. Isokpehi, Yogesh S. Shouche, Bayatigeri Santhakumari, Chiaka I. Anumudu

**Affiliations:** 1 Cell Biology and Genetics Unit, Department of Zoology, University of Ibadan, Ibadan, Nigeria; 2 Centre for Materials Characterization, CSIR-National Chemical Laboratory, Pune, India; 3 Parasitology Unit, Department of Zoology, University of Ibadan, Ibadan, Nigeria; 4 Department of Biological Sciences, Elizade University, Ilara-Mokin, Nigeria; 5 Department of Radiology, University of Ibadan, Ibadan, Nigeria; 6 College of Science, Engineering and Mathematics, Bethune-Cookman University, Daytona Beach, Florida, United States of America; 7 National Centre for Microbial Resources, National Centre for Cell Science, Pune, India; George Washington University, UNITED STATES

## Abstract

**Background:**

Metabolic fingerprinting analysis can offer insights into underlying reactions in a biological system; hence it is crucial to the understanding of disease pathogenesis and could provide useful tools for discovering biomarkers. We sought to examine the urine and plasma metabolome in individuals affected by urogenital schistosomiasis and its associated-bladder pathologies.

**Methodology:**

Blood and midstream urine were obtained from volunteers who matched our inclusion criteria among residents from Eggua, southwestern Nigeria. Samples were screened by urinalysis, microscopy, PCR and ultrasonography, and categorised as advanced (urogenital schistosomiasis associated-bladder pathologies), infection-only (urogenital schistosomiasis alone) and controls (no infection and no pathology). Metabolites were extracted and data acquired with ultra high-performance liquid chromatography coupled with Thermo Q-Exactive orbitrap HRMS. Data was analysed with MetaboAnalyst, Workflow4Metabolomics, HMDB, LipidMaps and other bioinformatics tools, with univariate and multivariate statistics for metabolite selection.

**Principal findings:**

There were low levels of host sex steroids, and high levels of several benzenoids, catechols and lipids (including ganglioside, phosphatidylcholine and phosphatidylethanolamine), in infection-only and advanced cases (FDR<0.05, VIP>2, delta>2.0). Metabolites involved in biochemical pathways related to chorismate production were abundant in controls, while those related to choline and sphingolipid metabolism were upregulated in advanced cases (FDR<0.05). Some of these human host and *Schistosoma haematobium* molecules, including catechol estrogens, were good markers to distinguish infection-only and advanced cases.

**Conclusions:**

Altered glycerophospholipid and sphingolipid metabolism could be key factors promoting the development of bladder pathologies and tumours during urogenital schistosomiasis.

## Author summary

Obtaining specific molecules having a strong association with a disease condition i.e. biomarkers, is usually a major step to developing new modes of therapy or diagnosis for the disease. In this study, samples from individuals with *S*. *haematobium* infection, some of whom had developed bladder pathologies, were compared to controls. From the blood and urine samples analysed by mass spectrometry, we highlight important human and *Schistosoma haematobium* metabolites, small molecules smaller in size than most proteins, which associate strongly with individuals having schistosomiasis induced-pathology or schistosomiasis alone. This report of the potential biomarkers will also add to the current understanding of the molecular events leading to schistosome associated-bladder cancer.

## Introduction

Among the most prominent neglected tropical diseases (NTDs) is schistosomiasis, a helminthic disease caused by *Schistosoma* spp. Its urinary form, caused by *S*. *haematobium* and known as urogenital or urinary schistosomiasis, is widespread in Africa and the Middle East. In chronic cases, infected persons may experience abdominal pain, enlarged liver, paralysis, granuloma formation, blood in the urine and the risk of early onset and aggressive bladder cancer [1]. A population of more than 200 million in different countries is at risk of schistosomiasis [1] and more than 100 million are said to be affected by urogenital schistosomiasis [2]. Several studies in different parts of Nigeria have reported moderate to high prevalence of urogenital schistosomiasis [3,4]. There is as yet no effective vaccine, and there are some reports of drug resistance due to over-reliance on praziquantel, the major drug in use [1,5], therefore there is a continuous search for molecular targets for the development of vaccines and chemotherapeutics.

Urogenital schistosomiasis has been associated with different forms of bladder pathologies in Nigeria and with bladder tumours in parts of Africa [6,7]. The molecular intricacies involved in the development of bladder tumours during urogenital schistosomiasis are not clearly defined, and the tumours are usually preceded by abnormal morphologies or pathologies especially in the bladder. Recent studies on the mechanisms of tumour development in schistosomiasis have highlighted the role of estrogen-related molecules from the parasite, as such molecules were found in many urine samples from infected persons in Angola [8,9,10]. Also, it was recently shown that an inflammation-regulatory microbiome could play important roles in the maintenance or development of bladder pathologies during infection [11].

Analysing the metabolome, the milieu of compounds in a body fluid, gives a comprehensive idea about the internal body reactions and its products [12]. This can be influential in biomarker discovery, diagnosis of disease and health conditions, and offer insights into disease mechanisms [13]. Indeed, it has led to efforts such as the Human Serum Metabolome and the Human Urine Metabolome projects [14]. Given the pathology of urogenital schistosomiasis, a deep understanding of the metabolome is important.

The aim of the present study is to examine the metabolome features in urine and plasma samples from persons infected with urogenital schistosomiasis and related pathologies in a rural population in Nigeria; and offer insights into host-parasite interaction and induction of bladder pathologies during urogenital schistosomiasis.

## Methods

### Ethics statement

The study protocol was approved by the University College Hospital/University of Ibadan Review Committee, as well as the Ogun State Ministry of Health. Ethical considerations were reported earlier [11]. Briefly, all adult participants were recruited into the study after giving written informed consent. Participants were informed of the purpose of the study, the health risks associated with the sample collection methods, and the potential benefits to public healthif biomarkers were to be discovered. Interviews and questionnaires were administered in the local language.

### Study participants and screening for urogenital schistosomiasis and pathologies

Participants were recruited from Eggua community in Ogun State, southwestern Nigeria. Sampling was carried out between December 2014 and June 2015. Sampling procedures and detection of *S*. *haematobium* infection and bladder pathologies were carried out as reported earlier [11]. Samples were immediately anonymised and aliquoted for microscopy. An aliquot of samples was immediately kept in ice chest and transported in dry ice to the laboratory, where they were kept at -80°C prior to analysis.

Participants were interviewed to obtain information on demographics and lifestyle. They provided blood samples from which plasma was isolated and midstream clean-catch urine samples in the morning hours upon instructions. Urine microscopy and PCR were used to confirm infection status. Bladder scans were carried out with TitanUltraSystem (Sonosite,USA) by a radiologist. No confirmed bladder cancer cases were detected but various forms of pathologies (abnormal morphologies) were observed; hence, samples were grouped based on presence of infection with pathologies (advanced), infection without pathologies (infection-only), pathologies without infection (pathology-only) and controls (no infection or pathology).

### LC-MS and bioinformatics analysis

Both urine and plasma samples were prepared using chilled methanol:water mixture (4:1), following previous methods [15].An Accela ultra high-performance liquid chromatography (UHPLC) system (ThermoFisher, USA), coupled online via heated electrospray ionization source (HESI) to a mass spectrometer was employed for non-targeted metabolomics profiling. Separation was achieved with 150mm x 2.1mm, 1.9μ HypersilGold column with a 5μl injection volume. The temperature of column oven was set at 40°C and the sample manager was maintained at 4°C. Gradient elution was performed using 0.1% formic acid in water (A) and acetonitrile (B) as mobile phase after modification and optimization of previous methods [9], and the elution was run witha mobile phase gradient of 0-6min, 100% A; 6-8min, linear gradient from 100% to 80% A; 8-12min, linear gradient from 80% to 40% A; 12–14min, linear gradient from 40% to 70% A; 14-16min, linear gradient from 70% to 80% A; 16-20min, linear gradient from 80% A to 100% B. The column was washed between each sample for stability and to eliminate any carry-overs. The flow rate was 0.35 ml/min.MS acquisition was performed on the Q-Exactive orbitrapmass spectrometer (ThermoFisher, MA, USA) operated in positive and negative electrospray ionization (ESI) modes. The sample sequence was set to random and samples were run in triplicates. Each sample type and ESI mode were run in a batch. In the ESI+ mode, the MS spray voltage was 4.2 KV while it was 3.6 KV in the ESI− mode. The capillary temperature was set at 300°C and probe heating temperature at 320°C with the sheath gas at 45 arbitrary units. For ESI+ and ESI− mode, the aux gas was set at 5 and 12 arbitrary units respectively. The tube lens was set to 50V and the mass scan range was set from 70 to 1000 m/z. The resolution of the orbitrap was set at 70,000. Both ESI modes were used to analyse urine and plasma samples (Table 1), but forty-two of the urine samples were analysed in the negative run due to inadvertent loss of samples.

**Table 1:** Classification of participants in metabolome study in Eggua, Nigeria.

Sample Categories	Number (%)
Control	46 (41.4)
Infected	65 (58.6)
	111
Advanced	32(28.8)
Pathology-Only	19(17.1)
Infection-Only	32(28.8
Controls	28(25.2)
	111
Female	48(43.2)
Male	63(56.8)
	111

Advanced: infection + abnormal bladder morphologies; Pathology-only: abnormal bladder morphologies; Infection-only: urogenital schistosomiasis.

### Metabolite processing and identification

Profile mode raw data were converted to centroid mode mzXML files with MSConvert and subjected to XCMS and CAMERA for pre-processing [16,17]. Peaks were identified with the xcmsSet algorithm using the centwave method, a mass tolerance of 3 ppm, and peakwidth range between 10–50 seconds. Peaks were matched (bandwidth = 5, mzwid = 0.015), retention time aligned using obiwarp method, peaks were regrouped and filled. They were annotated using xsAnnotate, groupFWHM (perfwhm = 0.6), findIsotopes (mzabs = 0.01), groupCorr(cor_eic_th = 0.75), and findAdducts parameters. The Workflow4Metabolomics galaxy server (https://galaxy.workflow4metabolomics.org/) [18] and MetaboAnalyst (http://www.metaboanalyst.ca) [19] were also used for processing and identification. Data was filtered (relative standard deviation) and normalized. Identification of metabolites were at least Level 2 identification. Accurate mass, spectral library match (low level MS/MS spectra), retention time, annotations (adduct, isotope combinations), and biological context (organism and type of body fluid of previous identification) were all used in comparison to available data with similar analytical procedures in The Human Metabolome Database [14], LipidMaps and Metlin database [20], allowing for molecular weight tolerance of 0.5Da. The naming of the metabolites are putative and metabolite family name is provided when more than one match occurs.

### Statistical and biomarker analysis

Metabolite features that varied significantly between sample groups were evaluated with Mann Whitney (two groups) or Kruskal-Wallis (more than two groups) tests with False Discovery Rate (FDR) correction, and inter-group separation based on the features were modeled with Partial Least Square Discriminant Analysis(PLSDA) and Principal Component Analysis.Analysis of feature importance for selection was carried out with PLSDA, Significance Analysis of Microarrays (and Metabolites) SAM and RandomForest trees [21, 22, 23]. Selected features were evaluated for use as biomarkers using multivariate ROC curve analysis [24] with logistic regression models.

## Results

Study information and data have been submitted to Metabolomics Workbench (study ID ST000934). Interviews revealed that the diet was essentially uniform, with virtually all participants recalling their routine diet as a strong starch base mixed with local vegetable. Samples were classified into four groups: the advanced group defined as urogenital schistosomiasis infection and associated-bladder pathologies (including irregular shape, bladder mass and localized thickening); the pathology-only group defined as bladder pathology with no indication of urogenital schistosomiasis infection; the infection-only group; and controls defined as persons with no infection or pathology. Additionally, for analytical purposes, samples were also grouped as infected and non-infected.

### Marked differences exist in metabolome features in urogenital schistosomiasis associated-bladder pathologies, urogenital schistosomiasis infection and controls

**Fig 1:**
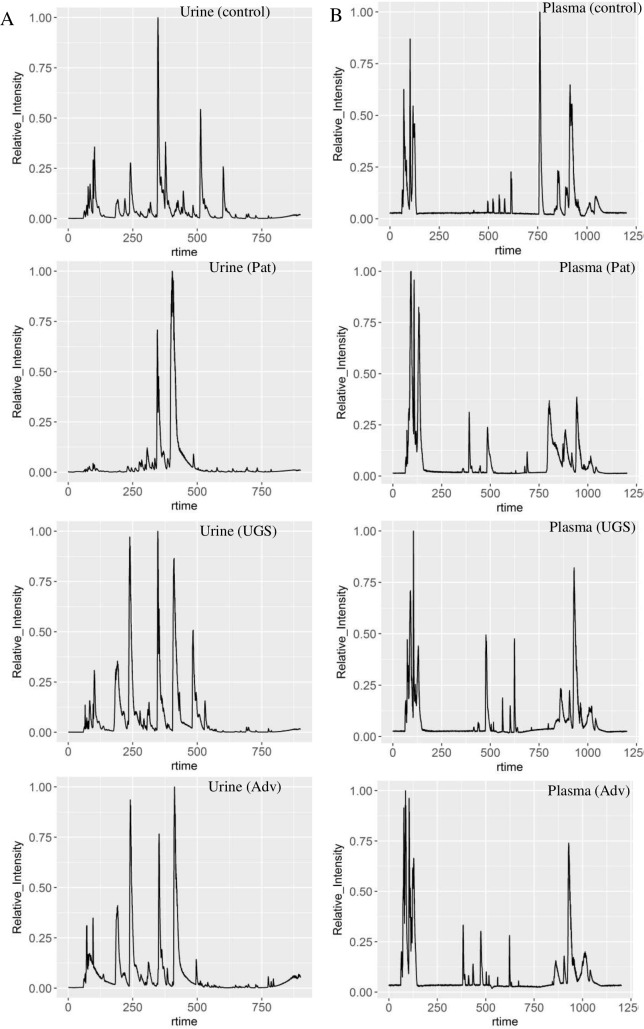
Total ion chromatogram of representative sample in healthy volunteers, and participants with urogenital schistosomiasis (UGS) and urogenital schistosomiasis induced-bladder pathology (Adv) in Eggua, Nigeria. (A) urine and (B) plasma samples.

There were differences in LC-MS features of urine and plasma samples from healthy controls, urogenital schistosomiasis and urogenital schistosomiasis induced-bladder pathologies (Fig 1A and 1B), especially from 6.5 to 12 minutes in urine samples (Fig 1A). The differential features, after univariate analysis (p<0.01), were subsequently filtered using FDR correction (FDR<0.01) (S1 File). In pairwise comparison of the study groups, the features which differentiate induced-bladder pathologies (advanced) from infection-only were far fewer in both modes and sample types than those which differentiate advanced from pathology-only (S2 File). Pathway analysis of the peaks detected is presented in S3 File. The number of the filtered significant features (FDR<0.01, fold change >2) was different depending on whether ionization mode was positive or negative. For plasma, among the four sample groups, 26% (2334) of the detected features were significantly different in the ESI negative mode, and 15% (983) in the positive mode (FDR<0.05). When plasma samples were simply grouped into two, infected and non-infected, 18% (1562) of the detected features were significantly different in the negative mode, and 4% (237) of the features detected in positive mode were significantly different (FDR<0.05). In urine samples, 648 (10%) differentiated the four groups in negative mode (FDR<0.05). When urine samples were grouped into two, infected and non-infected, 834 (13%) differentiated infected and non-infected in negative mode; and 891(7%) differentiated the same groups in positive mode.

**Fig 2:**
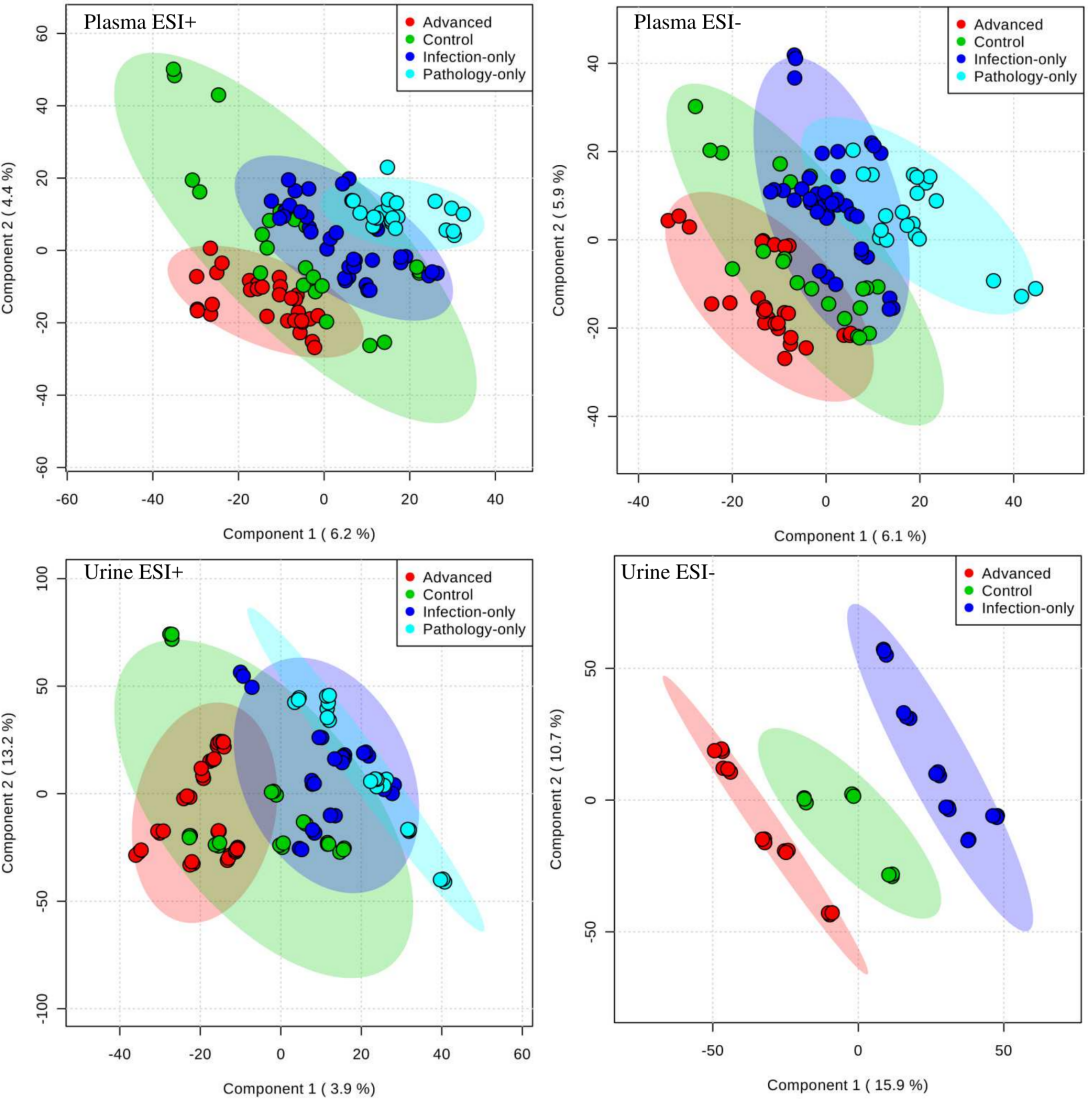
Urine and plasma samples have different separation patterns in different states of urogenital schistosomiasis and induced pathologies. Separation patterns were drawn with PLSDA score plots of mass spectral data. Spectral data was captured for urogenital schistosomiasis induced-pathology cases (Advanced), urogenital schistosomiasis alone (Infection-only), pathology with no detectable urogenital schistosomiasis (Pathology-only) and Controls. The plots show that statistically, the various study groups can be defined independently using their metabolite component. ESI is electrospray ionization.

**Fig 3:**
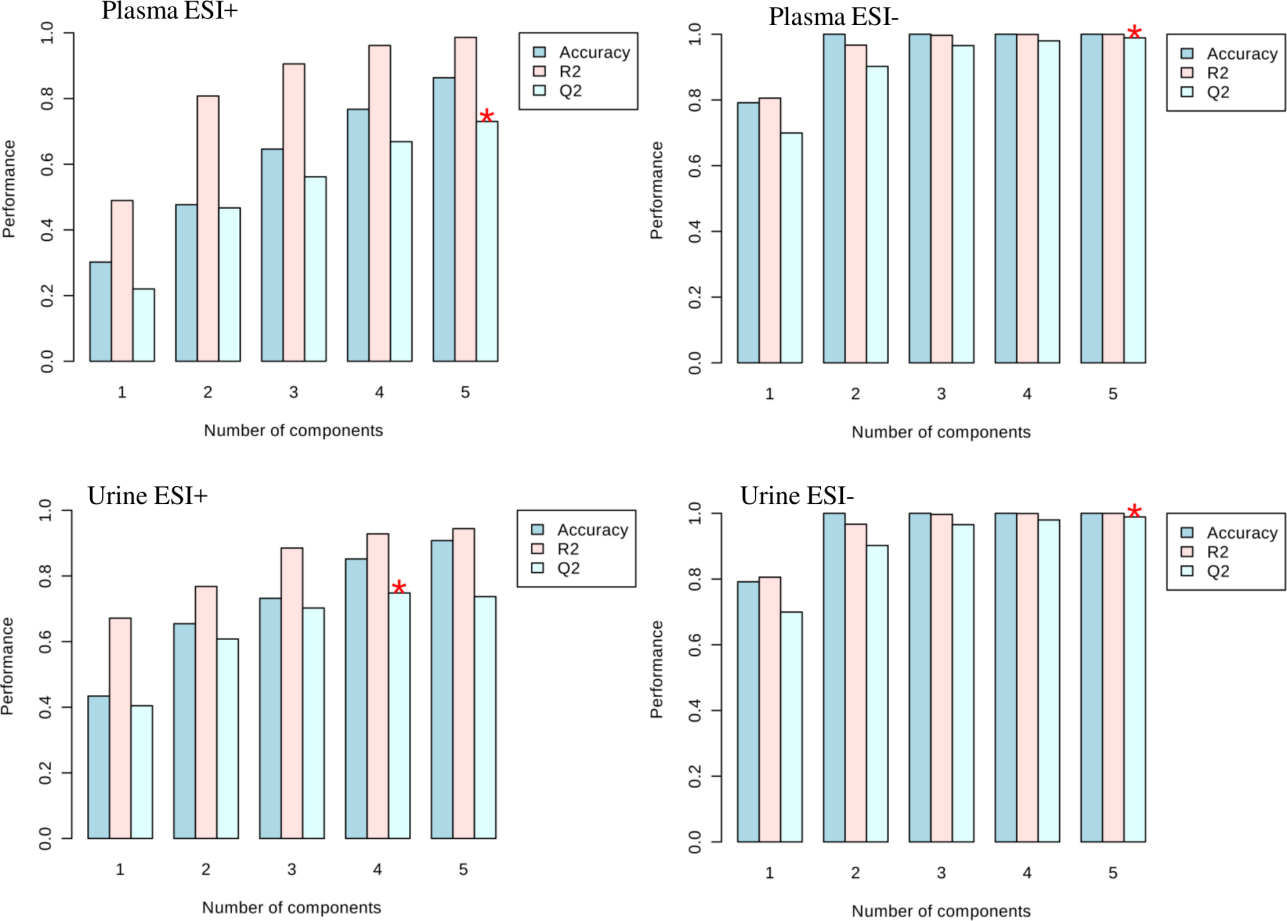
Discrimination of urine and plasma samples according to different states of urogenital schistosomiasis and induced pathologies can be validated. Cross validation plots of mass spectral data were drawn, with data capture for urogenital schistosomiasis induced pathology cases (Advanced), urogenital schistosomiasis alone (Infection-only), pathology with no detectable urogenital schistosomiasis (Pathology-only) and controls. The plots show that PLSDA discrimination were valid. ESI is electrospray ionization.

For multivariate analyses, using spectral data, PCA and PLSDA were performed and 95% confidence ellipses drawn. Both methods showed a trend of inter group axes separation, but as may be expected, PLSDA gave better axes separation than PCA (Fig 2). For PLSDA, Q^2^ validation was highest in urine samples in negative mode (> 0.99) and least for plasma in positive mode (0.8) (Fig 3). The scores plots of the PLSDA model also show that the inter-group discrimination (and therefore, the choice of representative metabolites for the various groups) could be maximized with several distinct metabolome features (Fig 2).

### Some differential metabolite peaks in urogenital schistosomiasis associated-bladder pathologies and urogenital schistosomiasis infection could be strong biomarkers

To select the most important metabolites in the high dimensional metabolomics data, PLSDA models, Significance analysis of Microarrays and Metabolites (SAM) and RandomForest (RF) trees were utilized. For SAM, a minimal false positive value (≤ 1) and relatively high delta (delta>2) were set in order to obtain accurate measurement of differentially expressed features. Also, the metabolite features were subjected to Random Forest’s algorithm, using 500 trees and 7 predictors, to determine the metabolites with the most important contribution. The highest RF misclassification occurred with plasma samples, with Out of the Bag (OOB) error of 0.0631. For PLSDA models, Variable Importance in Projection (VIP) scores were set at >2.

As a result of variation in the top features selected by the three algorithms, metabolites which were eventually selected for biomarker evaluation were chosen systematically. Biomarkers are expected to be robust in order to fit varying conditions. Hence, the goal of the systematic choice was to take advantage of overlapping top-ranked metabolites from three algorithms from a pool of metabolites detected in both positive and negative modes. First, metabolites from both ESI modes were combined, then those that were (1) significantly different among all the four study groups (FDR<0.05), (2) significantly different between infection-only and advanced cases (FDR<0.05), and (3) ranked high in two of three multivariate feature selection methods (SAM delta>3, PLSDA VIP score >1.5 or RF rank <200) were chosen (Tables 2 and 3). Several of the highly differential metabolites have no known matches in the Human Metabolome Database and are probable molecules produced by *S*. *haematobium* during urogenital schistosomiasis infection (Table 3). The suitability of the selected features as biomarkers of urogenital schistosomiasis associated-bladder pathology and urogenital schistosomiasis were then analysed with Receiver Operating Characteristics (ROC) curve analysis. The performance characteristics of these probable biomarkers using logistic regression algorithm are presented in Table 4.

**Table 2:** Identified human metabolites strongly associated with different categories of urogenitalschistosomiasis (fdr<0.05, delta>2, VIP score >1.5).

Metabolite/ Retention Time (min)	Putative Identity	Abundance in	Fold increase (compared to other groups)	Biochemical pathway	Ionisation; Sample
269.1900/9.4; 287.2002/9.4	Estrogen/Androgen precursors	Control	2.3–2.4	steroid biosynthesis &degradation	Positive;urine
383.2063/11.1	TLB	Control	2.5	lipid metabolism	Positive;urine
209.0662/1.4	Fucosylated sugar	Control	<2–2.8	Fructose and mannose metabolism	Negative;plasma
367.1588/10.2	modified estradiol/Testosterone	Advanced	<2–2.3	steroid hormone synthesis	Negative;plasma
180.0655/7.1	Adrenochrome O-quinone	Infection-only	2.1–3.3	Parasite-modified host metabolite	Positive;plasma
267.0741/9.2	Indolylacryloylglycine	Advanced/Infection-only	2.5	Tryptophan metabolism	Positive;urine
785.5880/16.7	N-Glycoloylganglioside GM2	Advanced	3.4–5.5	sphingolipid metabolism	Positive;plasma
807.5723/17.1	Phosphatidylcholine (PC)	Advanced	2.2–4.3	Choline metabolism in cancer/glycerophospholipid metabolism	Positive;plasma
806.5689/17.1	Phosphatidylethanolamine (PE	Advanced	<2–3.3	Choline metabolism in cancer/glycerophospholipid metabolism	Positive;plasma
512.2999/13.6	LysoPC(14:0)/1HGPE	Advanced	2.0–2.8	Choline metabolism in cancer/glycerophospholipid metabolism	Negative;plasma
180.0541/7.6	3-Succinoylpyridine	Infection only	2.9–4.0	Parasite-modified host metabolite	Negative;plasma

Metabolite identification was through accurate mass, spectral library match, retention time, adduct/isotope combinations and biological context. Advanced—urogenital schistosomiasis induced-pathology cases; Infection-only–urogenital schistosomiasis alone, Pathology-only—pathology with no detectable urogenital schistosomiasis; Control–no infection or pathology. HGPE—1-Heptadecanoylglycerophosphoethanolamine; TLB—12-Oxo-20-trihydroxy-leukotriene B4.

**Table 3:** Probable novel *S*. *haematobium* molecules as potential biomarkers of urogenital schistosomiasis associated bladder pathology (advanced) and urogenital schistosomiasis (infection-only).

Metabolite/RT	ESI mode	Remarks on Identity	Disease status	Fold increase	Sample
335.0448/4.6	Negative	Parasite Catechol	Advanced	10.5	Urine
626.3559/10.2	Negative	Parasite-derived	Advanced	4.8/22.2	Plasma/Urine
397.1606/3.7	Positive	Parasite-derived	Advanced	<2.0	Urine
352.8331/1.5	Negative	Parasite-derived	Advanced	4.7	Urine
510.1275/5.88	Negative	Parasite benzoquinolone/PAH	Advanced	7.0	Urine
242.0221/5.7	Negative	1-Nitro-5,6-dihydroxy-dihydronaphthalene	Infection-only	13.3	Urine
323.0712/5.8	Negative	Parasite-derived	Infection-only	4.5	Urine
152.0707/5.3	Positive	Parasite catechol/benzoquinone/aminobenzenoid	Infection-only	4.3	Urine
356.2795/13.38	Positive	Parasite-derived	Infection-only	4.3	Urine
192.9806/2.8	Negative	Parasite-derived	Advanced	6.3	Urine
206.9965/3.9	Negative	Parasite-derived	Advanced	5.3	Urine
293.0492/2	Negative	Parasite-derived	Advanced	2.5	Plasma
263.0286/7.63	Negative	Parasite-derived	infection-only	2.3	Plasma

RT-Retention Time in minutes. Fold increase is in comparison to advanced or infection-alone. Parasite-derived indicates a metabolite not present in any known database. Metabolite identification was through accurate mass, spectral library match, adduct/isotope combinations and biological context.

**Table 4:** Putative biomarkers of urogenital schistosomiasis and associated bladder pathology.

Metabolite m/z	Putative Identification	Stage marker	Sample	AUC 1	AUC 2	ESI mode	Retention Time
512.2999	LysoPC	Advanced	Plasma/Urine	0.8	0.85	Negative	13.6min
180.0541	SP	Infection-only	Plasma/Urine	0.74	0.78	Both	7.6min
335.0448	Catechol	Advanced	Urine	0.8	1	Negative	4.6min
242.0221	DDN	Infection-only	Urine	0.77	0.87	Negative	5.7min
269.1507	CEQ	Infected	Urine	0.84	0.78	Negative	8.98min
228.0876	CEQ	Infected	Urine	0.95	0.85	Negative	5.58min
204.1319	8ODG	Infected	Urine	0.81	0.8	Negative	8.38min
369.0832	CEQ	Infected	Urine	0.82	0.83	Negative	6.37min
807.5723	PC	Advanced	Plasma	0.66	0.73	Positive	17.1min
806.5689	PE	Advanced	Plasma	0.69	0.78	Positive	17min
785.5880	GGM	Advanced	Plasma	<0.6	0.8	Positive	17min

AUC1: Area under Cover score for the metabolite as biomarker of urogenital schistosomiasis; AUC2: Area under Cover score for the metabolite as biomarker to distinguish urogenital schistosomiasis associated-bladder pathology or infection alone; AUC, an ROC statistic for biomarker testing; was calculated with Monte Carlo cross validation; CEQ- catechol estrogen quinine; 8ODG- 8 oxodG or 8-hydroxy-2’-deoxyguanosine; SP– 3-succinoylpyridine; DDN -1-Nitro-5,6-dihydroxy-dihydronaphthalene; PE-phosphatidylethanolamine; PC-phosphatidylcholine; GGM-N-Glycoloylganglioside GM2. Metabolite identification was through accurate mass, spectral library match, retention time, adduct/isotope combinations and biological context.

Of interest in the current study were the *S*. *haematobium* catechol estrogen and related molecules identified previously in Santos *et al*. (2014) and Gouveia *et al*. (2015), in which urine was examined in ESI negative mode only. In the current study, 11metabolites appeared to match those highlighted in the two aforementioned studies (Table 5).

**Table 5:** Probable *S*. *haematobium* estrogen related metabolites in urine of study participants in Eggua, Nigeria.

Probable SES metabolites (m/z)	Retention Time (min)	Previous marker identification	Class of metabolite
305.1264	10.15	Santos *et al*. (2014)	CEQ
269.1507*	8.98	Santos *et al*. (2014)	CEQ
481.2487	9.55	Santos *et al*. (2014)	CEQ
228.0876*	5.58	Gouveia *et al*. (2015)	CEQ
246.0161	5.90	Gouveia *et al*. (2015)	CEQ
274.0031	4.15	Gouveia *et al*. (2015)	8ODG
190.0134	6.90	Gouveia *et al*. (2015)	8ODG
204.1319*	8.38	Gouveia *et al*. (2015)	8ODG
265.1061	8.65	Gouveia *et al*. (2015)	8ODG
285.1712	9.65	Gouveia *et al*. (2015)	8ODG
369.0832*	6.37	Gouveia *et al*. (2015)	CEQ

SES- *S*. *haematobium* estrogen related metabolite; CEQ- catechol estrogen quinone

8ODG- 8 oxodG or 8-hydroxy-2′-deoxyguanosine

*Highly significant biomarker FDR<0.05

## Discussion

In this study, LC-MS was used to examine urine and plasma metabolites in samples from people with different states of urogenital schistosomiasis, and those without infection (controls). Important metabolites were putatively identified by peak annotations, from searching metabolome databases and mass spectral matching. In general, in both urine and plasma a large number of chromatographic peaks were detected and hence, a large number of metabolites. The large number of differential metabolites among study groups (S1 and S2 Files) which were statistically significant indicates that there were many peaks that could be explored for biomarker use. Nevertheless, a higher number of unique metabolites were found in urine compared to plasma, probably because the parasite inhabits the bladder environment.

It is clear from the data that abnormal lipid regulation in the human host is important in both urogenital schistosomiasis infection and urogenital schistosomiasis associated-bladder pathologies as several of the most dysregulated metabolites from the metabolome were lipids (Table 2). Many proteins, including those involved in developing pathological conditions, interact with lipids of the cell membranes and these protein–lipid interactions are susceptible to modifications [25]. Thus, alterations in lipid levels observed in this study would definitely lead to changes in protein activity. A similar situation of altered lipid metabolism has been associated with the development of cardiovascular pathologies, such as hypertension, atherosclerosis, coronary heart disease and thrombosis, as well as tumours [26,27]. There is also evidence that the changes in phospholipids may occur before morphological changes in tumours [28], and there are attempts to target them with drugs [25]. Given that no bladder carcinomas were recorded among participants, we suggest that specific lipid metabolites highlighted in this study may serve as early warning metabolites prior to the development of bladder cancer, during chronic urogenital schistosomiasis. They may be useful as early diagnostic markers or therapeutic targets.

Among the most downregulated metabolites in urogenital schistosomiasis and urogenital schistosomiasis- associated bladder pathology cases were human steroid hormone precursors (Table 2) and this observation is supported by the number of metabolic pathway hits (S3 File). These steroid precursors are required to produce estrogens, estradiol and testosterone. Thus, the finding in this study is that host steroids are much reduced as a result of urogenital schistosomiasis. Given an earlier study [8] which found increased infertility occurrence along with urogenital schistosomiasis, one of the observations of the present study i.e. low level of host sex hormone precursor in infection, provides more insight into a possible mechanism by which urogenital schistosomiasis causes infertility. In the study, Santos *et al*. [8] found that 17 of 29 Angolan women infected with urogenital schistosomiasis had self-reported infertility, compared to 8 of 24 women infected with urogenital schistosomiasis who showed no signs of infertility. It had been suspected that hormonal imbalances may be a factor in this form of infertility [10]. To the best of our knowledge, this is the first study to provide evidence of significant reduction in the levels of human sex hormone precursors in urogenital schistosomiasis infection (Table 3) and its associated bladder pathologies.

Furthermore, several of the important metabolites which distinguished infection were those ostensibly produced by the parasite *S*. *haematobium*, and which could not be identified on currently curated databases such as HMDB, Metlin or LipidMaps. Eleven of these were matched to the catechol estrogens associated with infection in [8] and Gouveia *et al*. [9] (Table 5), although conditions of LC-MS analysis were different. However, the presence of trace amounts of such metabolites in some non-infected persons is an indication that better diagnostic tools are still needed for urogenital schistosomiasis and that its burden in Nigeria may be underestimated. Based on the current study (Table 5), four of these 11 estrogen-related parasite molecules detected, m/z 269, 228, 204 and 369, were significant enough to be considered as biomarkers. In the report of Gouveia *al*. [9], urine samples from participants with urogenital schistosomiasis-associated hyperplasia, metaplasia squamous or urothelial carcinoma were analysed, but the current study involved participants with healthy controls, ordinary infection or infection with the bladder pathologies which precede carcinoma, rather than confirmed carcinoma cases. Therefore, it is likely that the full repertoire of the schistosome catechol estrogens is abundant only in the matured/developed carcinoma cases. In addition, the differences in parts of the analytical conditions used in the study may result in differences in results. Another parasite molecule, m/z 335, a putative catechol, proved to be a good biomarker of urogenital schistosomiasis associated bladder pathology (Table 3). Many of these *S*. *haematobium* molecules in the current study will require further validation research which would involve comparison with standards and targeted tandem MS^n^ in order to determine their structure or whether they are modified forms; although in this study, the peak annotations and chemical identity have been revealed.

Because high levels of potential parasite estrogen-related molecules and low levels of host estrogen-related molecules were observed in the present data, we suggest that estrogen metabolism could be a key influential reaction in host-parasite relationship during urogenital schistosomiasis and could be important as the infection progresses into tumour. Considering the evidence from the current study and that from previous studies, we suggest that *S*. *haematobium* adult worm infection reduces human sex hormone availability either by utilising host steroid hormones or blocking its utilisation or production; and therefore, the worm produces related molecules. This proposed hypothesis is strengthened by earlier research [29] which showed that flatworms such as *Taenia spp* or hookworms may depend extensively on host sex steroids for growth. Such dependence could be expected to lead to lower levels of the host steroids.

Among the most upregulated metabolites in urogenital schistosomiasis and urogenital schistosomiasis associated bladder pathology cases were molecules belonging to catechols, cyclic aromatic hydrocarbons, benzenoids or quinones. These included many parasite and some host metabolites (Table 4). Abnormal levels of such metabolites are known to be involved in carcinogenesis.

There was an abundance of two related glycerophospholipids in advanced cases and to a lesser extent, in infection-only cases. Also, glycerophospholipid metabolism had high number of metabolic pathway hits (S3 File). High levels of phosphatidylcholine (PC) and phosphatidylethanolamine (PE) were found strongly associated in urogenital schistosomiasis associated-bladder pathology (Advanced) cases (Table 4). PE may be converted to PC by phosphatidylethanolamine-N-methyltransferase [30]. PC is formed from phosphocholines and catabolised back to phosphocholines. Increased levels of phosphocholines are associated with proliferation, and there are reciprocal interactions between oncogenic signalling and phosphocholine metabolism [31]. Increase in PCs, one of the major forms in the alteration of choline metabolism, involving specific phospholipases, transporters, kinases, was recently shown in induction of cancer [32], including colorectal cancers and non-small-cell lung cancer [30]. Due to oxidative stress in a tumour microenvironment, PE becomes highly expressed on endothelial cells as they are redistributed from the inner to the outer membrane leaflet [33]. It is suggested that increased PC and PE in bladder endothelial cells is one of the mechanisms for cancer induction in chronic urogenital schistosomiasis.

Gangliosides are glycosphingolipids containing sialic acid found mainly in the plasma membrane and having functions in cell recognition and signalling. From our data, N-Glycoloylganglioside GM2 (GGM), a ganglioside, was abundant in advanced cases (Table 2). GM2 gangliosides are over- expressed and abundant in different forms of carcinoma, including melanoma, neuroblastoma and breast carcinoma; they promote T cell dysfunction and have been utilised in vaccine trials [34]. There are also reports that these gangliosides are involved in pathological processes, because they can be receptors for viruses, toxins, and autoantibodies, and that they can suppress availability of innate and adaptive immune molecules [35]. In this study, GGM were found in bladder pathologies with no tumours observed; it was shown in breast cancer research by Azordegan et al. [28] that changes in phospholipids may occur before morphological changes in tumours. This study is the first to report a ganglioside strongly associated with urogenital schistosomiasis associated-bladder pathology.

In a similar vein, increased levels of benzenamines, putatively identified as Adrenochrome and 3-Succinoylpyridine, associated strongly with urogenital schistosomiasis infection alone. The abundance of these molecules is biologically relevant. Adrenochrome (also Adrenochrome-O-quinone; AQ), is a toxic quinone metabolite of catecholamines, specifically epinephrine. It is formed as a result of oxidation activities, is neurotoxic and has psychotomimetic properties [36]. Like other such quinones, AQ is capable of forming reactive oxygen species with pathological consequences. Glutathione transferases (GSTs) may prevent pathologies by catalysing the formation of glutathione conjugates of o-quinones [36].AQ abundance may be affected by other factors. GSTs attempt to scavenge free radical forming agents to prevent pathologies or cancer and some free radical species inhibit GST to prevent this. Polymorphisms in GST determine the efficiency of these processes and also determine the susceptibility to cancer. Hence, the efficiency may actually reduce the formation or availability of these agents, but further research will be needed to confirm this. However, abundance of AQ in infection-only cases would indicate that there is an increased amount of free reactive oxygen species and would therefore hasten bladder pathologies. It would also indicate inadequate activity, decreased activity or inhibition of GSTs. Such a decrease in GST activity due to *S*. *haematobium* infection was reported earlier [37].

3-Succinoylpyridine (3SP) a nicotine metabolite and by-product of N-nitrosamine formed by the action of cytochrome P450. N-nitrosamines (and especially N-nitrosodimethylamine, NDMA), nitrite and nitrate were detected in significant amounts in the urine of schistosomiasis patients in the studies [38] and the authors suggested they have roles in carcinogenesis. In the current study 3SP was found in abundance in infection-only cases; it is known to be a nicotine metabolite expected in the urine of tobacco smokers [39]. Participants in the current study did not indicate having a smoking history. 3SP is produced by hydroxylation of methylnitrosaminopyridylbutanone (NNK), a nitrosamine which was not among those reported by Mostafa et al. [38] to be in the urine of urogenital schistosomiasis patients. Thus, it has been suggested that quinones could be formed and N-nitrosamines are present in urogenital schistosomiasis infection [38]. This study is the first report of the abundance of Adrenochrome-o-quinone and NNK by-product 3SP in urogenital schistosomiasis.

Furthermore, a modified peptide, indolylacryloylglycine (IAG), was abundant in urogenital schistosomiasis associated-bladder pathology. IAG is suspected to be a by-product of tryptophan metabolism and glycine conjugation, a process which may or may not involve gut bacteria action [40]. IAG levels in urine were reported to be increased in muscular pathologies, autism, skin tuberculosis, and probably reduced in tumour [40,41] However, its use as a marker may be of limited value because its concentration varies seasonally, possibly due to higher solar radiation, and depending on age [41].

A putative naphthalene based compound, 1-Nitro-5,6-dihydroxy-dihydronaphthalene (DDN), was abundant in, and a putative biomarker of, infection-only cases. Napthalene is classified by the International Agency for Cancer Research as a 2B carcinogen because at proper doses, naphthalene metabolites show genotoxic and/or mutagenic activity [42]. Napthalene is normally expected as an environmental pollutant in air and sometimes water effluents, and chronic inhalation of naphthalene can induce respiratory tract tumours [43]. In a similar mechanism as other weak carcinogens such as estrogens and benzene, naphthalene is metabolically activated by cytochrome P450 (CYP) and metabolites formed react with DNA to form depurinating adducts [43]. In the KEGG database, several metabolites of naphthalene including dihydroxy-dihydronapthalenes are annotated in the metabolism of xenobiotics by CYP (map00980), but not DDN. Since the participants in this study live in a rural setting and relatively far from air pollutants such as naphthalene (although air pollution from a nearby cement factory cannot be ruled out), we suggest that just as estrogen-like molecules were found to be produced by *S*. *haematobium*, benzenoids and related molecules may also be produced by the parasite and metabolised by human CYP, leading to their activation.

A limitation of the current study is lack of complementary data such as a validation set of completely different samples and higher order MS^2^ or MS^3^ data. This would have further enhanced definitive identification status of all metabolites.

In summary, unique putative metabolites with potential value as biomarkers were identified in this study; when these metabolites are completely validated, the molecules and associated proteins could be further characterised and studied for future use as therapeutic or diagnostic targets, or in vaccine development.

## Supporting information

S1 File **Metabolite features dysregulated in humans with urogenital schistosomiasis infection and controls (non-infected) in (A) plasma and (B) urine.** Metabolites were normalized by sum, filtered using relative standard deviation and selected using fdr<0.05 and fold change>2. Samples were examined in negative (ESI-) or positive (ESI+) ionisation modes. Advanced—urogenital schistosomiasis induced-pathology cases; Infection-only–urogenital schistosomiasis alone, Pathology-only—pathology with no detectable urogenital schistosomiasis.
(XLSX)

S2 File Pairwise comparison of metabolite features dysregulated in human plasma and urine in stages of urogenital schistosomiasis infection with univariate analysis. Metabolites were normalized by sum, filtered using relative standard deviation and selected using FDR<0.001 and fold change>2. Samples were examined in negative (ESI-) or positive (ESI+) ionisation modes. Advanced—urogenital schistosomiasis induced-pathology cases; Infection-only–urogenital schistosomiasis alone, Pathology-only—pathology with no detectable urogenital schistosomiasis.
(XLSX)

S3 File Top significantly enriched metabolic pathways in urogenital schistosomiasis infection with the *Homo sapiens* metabolic model MFN (p<0.01) using peaks detected in (A) plasma samples using negative ionization mode (B) plasma samples using positive ionization mode, (C) urine samples in positive ionization mode and (D) urine samples in negative ionization mode.
(XLSX)

## References

[1] World Health Organization (WHO). Fact Sheets- Schistosomiasis. 2017. Available from http://www.who.int/mediacentre/factsheets/fs115/en/.

[2] Rinaldi G, Young ND, Honeycutt JD, Brindley PJ, Gasser RB, Hsieh MH. New research tools for urogenital schistosomiasis. Journal of Infectious Diseases 2015; 211:861–9. doi: 10.1093/infdis/jiu527 25240172 10.1093/infdis/jiu527 PMC4416124

[3] Anumudu CI, Alabi O, Oniya MO. Schistosome specific antibodies in individuals co-infected with malaria in Southwest Nigeria. Nigerian Journal of Parasitology 2012; 33(2): 133–139.

[4] Abdulkadir A, Ahmed MA, Abubakar BM, Yusuf SI, Imam MI, Sule AA, et al Prevalence of urinary schistosomiasis in Nigeria, 1994–2015: Systematic review and meta-analysis. African Journal Urology 2017; 23: 228–239. doi: 10.1016/j.afju.2016.11.004

[5] Wolstenholme AJ, Martin RJ. Anthelmintics–From Discovery to Resistance. International Journal for Parasitology: Drugs and Drug Resistance 2014; 4: 218–219. doi: 10.1016/j.ijpddr.2014.10.001 25516831 10.1016/j.ijpddr.2014.10.001 PMC4266783

[6] Onile OS, Awobode HO, Oladele VS, Agunloye AM, Anumudu CI. Detection of urinary tract pathology in some Schistosoma haematobium infected Nigerian adults. Journal of Tropical Medicine 2016; ID 5405207. doi: 10.1155/2016/5405207 10.1155/2016/5405207 PMC5011230 27635146

[7] Barsoum RS. Urinary Schistosomiasis: Review. Journal of Advanced Research 2013; 4(5):453–459. doi: 10.1016/j.jare.2012.08.004 25685452 10.1016/j.jare.2012.08.004 PMC4293885

[8] Santos J, Gouveia MJ, Vale N, Delgado ML, Goncalves A, et al Urinaryestrogen metabolites and self-reported infertility in women infected with Schistosoma haematobium. PLoS ONE 2014; 9(5): e96774 doi: 10.1371/journal.pone.0096774 24848950 10.1371/journal.pone.0096774 PMC4029575

[9] Gouveia MJ, Santos J, Brindley PJ, Rinaldi G, Lopes C, Santos LL, et al Estrogen-like metabolites and DNA-adducts in urogenital schistosomiasis-associated bladder cancer. Cancer Letters 2015; 359: 226–232. doi: 10.1016/j.canlet.2015.01.018 25615421 10.1016/j.canlet.2015.01.018

[10] Botelho MC, Alves H, Richter J. Estrogencatechols detection as biomarkers in schistosomiasis induced cancer and infertility. Lett Drug DesDiscov. 2017; 14(2): 135–138. 10.2174/1570180813666160720165057 PMC5179139 28018158

[11] Adebayo AS, Survayanshi M, Bhute S, Agunloye AM, Isokpehi RD, Anumudu CI, et al The microbiome in urogenitalschistosomiasis and induced bladder pathologies. PLoS Neglected Tropical Diseases 2017; 11(8): e0005826 doi: 10.1371/journal.pntd.0005826 28793309 10.1371/journal.pntd.0005826 PMC5565189

[12] Di Girolamo F, LanteI Muraca M and Putignani L. The role of mass spectrometry in the “omics” era. Current Organic Chemistry 2013;17: 2891–2905. doi: 10.2174/1385272817888131118162725 24376367 10.2174/1385272817888131118162725 PMC3873040

[13] Vernocchi P, Vannini L, Gottardi D, DelChierico F, Serrazanetti DI. Integration of datasets from different analytical techniques to assess the impact of nutrition on human metabolome. Frontiers in Cellular and Infection Microbiology 2012; 2:156 doi: 10.3389/fcimb.2012.00156 23248777 10.3389/fcimb.2012.00156 PMC3518793

[14] Wishart DS, Jewison T, Guo AC, Wilson M, Knox C, et al HMDB 3.0—The Human Metabolome Database. Nucleic Acids Res. 2013;41(D1): D801–7. 23161693 10.1093/nar/gks1065 PMC3531200

[15] Lu K, Abo RP, Schlieper KA, Graffam ME, Levine S, Wishnok JS. Arsenic exposure perturbs the gut microbiome and its metabolic profile in mice: an integrated metagenomics and metabolomics analysis. Environmental Health Perspectives 2014; 122(3): 284–291. doi: 10.1289/ehp.1307429 24413286 10.1289/ehp.1307429 PMC3948040

[16] Smith CA, Want EJ, O'Maille G, Abagyan R, Siuzdak G. XCMS: Processing Mass Spectrometry Data for Metabolite Profiling Using Nonlinear Peak Alignment, Matching, and Identification. Analytical Chemistry 2006; 78 (3): 779–787. doi: 10.1021/ac051437y 16448051 10.1021/ac051437y

[17] Kuhl C, Tautenhahn R, Böttcher C, Larson TR, Neumann S. CAMERA: An integrated strategy for compound spectra extraction and annotation of LC/MS data sets. Analytical Chemistry. 2012;84(1):283–289. doi: 10.1021/ac202450g 22111785 10.1021/ac202450g PMC3658281

[18] Giacomoni F, Le Corguillé G, Monsoor M, Landi M, Pericard P, Pétéra M, et al Workflow4Metabolomics: a collaborative research infrastructure for computational metabolomics. Bioinformatics. 2015;31(9):1493–5. doi: 10.1093/bioinformatics/btu813 25527831 10.1093/bioinformatics/btu813 PMC4410648

[19] Xia J, Wishart DS. Using MetaboAnalyst 3.0 for comprehensive metabolomics data analysis Current Protocols in Bioinformatics 2016; 55: 14101–141091. 10.1002/cpbi.11 27603023

[20] Tautenhahn R, Cho K, Uritboonthai W, Zhu Z, Patti G, Siuzdak G. An accelerated workflow for untargeted metabolomics using the METLIN database. Nature Biotechnology 2012;30: 826–828. doi: 10.1038/nbt.2348 10.1038/nbt.2348 PMC3666346 22965049

[21] Tusher VG, Tibshirani T, Chu G. Significance analysis of microarrays applied to the ionizing radiation response. Proc Natl Acad Sci. 2001; 98 (9): 5116–5121. doi: 10.1073/pnas.091062498 11309499 10.1073/pnas.091062498 PMC33173

[22] Barker W, Rayens W. Partial least squares for discrimination. J. Chemom. 2003; 17: 166–173.

[23] Ho TK. Random decision forests. Proceedings of the 3rd International Conference on Document Analysis and Recognition, Montreal. 1995 August 14–16; 278–282. Available from http://ieeexplore.ieee.org/document/598994/?reload=true.

[24] Zweig MH, Gregory C. Receiver-operating characteristic (ROC) plots: a fundamental evaluation tool in clinical medicine. Clinical Chemistry 1993; 39 (8): 561–577. 8472349

[25] Lladó V, López DJ, Ibarguren M, Alonso M, Soriano JB, Escribá PV, et al Regulation of the cancer cell membrane lipid composition by NaCHOleate: Effects on cell signaling and therapeutical relevance in glioma. Biochimica et Biophysica Acta 2014; 1838: 1619–1627. doi: 10.1016/j.bbamem.2014.01.027 24525074 10.1016/j.bbamem.2014.01.027

[26] Perona JS, Ruiz-Gutierrez V. Triacylglycerol molecular species are depleted to different extents in the myocardium of spontaneously hypertensive rats fed two oleic acid-rich oils. Am J Hypertens. 2005; 18: 72–80. doi: 10.1016/j.amjhyper.2004.11.012 15691620 10.1016/j.amjhyper.2004.11.012

[27] Perrotti F, Rosa C, Cicalini I, Sacchetta P, Del Boccio P, Genovesi D, et al Advances in lipidomics for cancer biomarkers discovery. International Journal of Molecular Sciences 2016; 17: 1992 doi: 10.3390/ijms17121992 10.3390/ijms17121992 PMC5187792 27916803

[28] Azordegan N, Fraser V, Le K, Hillyer LM, Ma DW, Fischer G. Carcinogenesis alters fatty acid profile in breast tissue. Mol Cell Biochem. 2013; 374: 223–232. doi: 10.1007/s11010-012-1523-4 23180247 10.1007/s11010-012-1523-4

[29] Miyashita H, Nakagawa H, Kobayashi K, Hoshi M, Matsumoto M. Effects of 17-estradiol and bisphenol a on the formation of reproductive organs in Planarians. Biol Bull. 2011; 220: 47–56. doi: 10.1086/BBLv220n1p47 21385957 10.1086/BBLv220n1p47

[30] Zinrajh D, Hörl G, Jürgens G, Marc J, Sok M, Cerne D. Increased phosphatidylethanolamine N‑methyltransferase gene expression in non‑small‑cell lung cancer tissue predicts shorter patient survival. Oncology Letters 2014; 7: 2175–2179. doi: 10.3892/ol.2014.2035 24932311 10.3892/ol.2014.2035 PMC4049682

[31] Glunde K, Bhujwalla ZM, Ronen SM. Choline metabolism in malignant transformation. Nat Rev Cancer 2015; 11(12): 835–848. 10.1038/nrc3162 PMC4337883 22089420

[32] Bagnoli M, Granata A, Nicoletti R, Krishnamachary B, Bhujwalla ZM, Canese R, et al Choline metabolism alteration: a focus on ovarian cancer. Front Oncol. 2016; 6:153 doi: 10.3389/fonc.2016.00153 27446799 10.3389/fonc.2016.00153 PMC4916225

[33] Stafford JH, Thorpe PE. Increased exposure of phosphatidylethanolamine on the surface of tumor vascular endothelium. Neoplasia 2011; 13: 299–308. 21472134 10.1593/neo.101366 PMC3071078

[34] Krengel U, Bousquet PA. Molecular recognition of gangliosides and their potential for cancer immunotherapies. Frontiers in Immunology 2014; 5:325 doi: 10.3389/fimmu.2014.00325 25101077 10.3389/fimmu.2014.00325 PMC4104838

[35] Daniotti JL, Lardone RD, Vilcaes AA. Dysregulated expression of glycolipids in tumor cells: from negative modulator of anti-tumor immunity to promising targets for developing therapeutic agents. FrontOncol. 2016; 5:300 doi: 10.3389/fonc.2015.00300 10.3389/fonc.2015.00300 PMC4703717 26779443

[36] Baez S, Segura-Aguilar J, Widersten M, Johansson A, Mannervik B. Glutathione transferases catalyse the detoxication of oxidized metabolites (o-quinones) of catecholamines and may serve as an antioxidant system preventing degenerative cellular processes. Biochem J. 1997; 324: 25–28. 9164836 10.1042/bj3240025 PMC1218396

[37] Sheweita SA, El-Shahat FG, Bazeed MA, El-Maati AMR, O’Connor PJ. Effects of Schistosoma haematobium infection on drug-metabolizing enzymes in human bladder cancer tissues. Cancer Letters 2005; 205: 15–21. 10.1016/j.canlet.2003.09.023 15036656

[38] Mostafa MH, Sheweita SA, O’Connor PJ. Relationship between schistosomiasis and bladder cancer. Clin Microbiol Rev.1999; 12: 97–111. 9880476 10.1128/cmr.12.1.97 PMC88908

[39] Felicia ND, Rekha GK, Murphy SE. Characterization of cytochrome P450 2A4 and 2A5 catalysed 4-(methylnitrosamino)-1-(3-pyridyl)-1-butanone (NNK) metabolism. Arch. BiochemBiophys.2000; 384,418–424. 10.1006/abbi.2000.2128 11368333

[40] Marklova E. Where does indolylacrylic acid come from? Amino Acids 1999; 17:401–413 10707769 10.1007/BF01361665

[41] Bull G, Shattock P, Whiteley P, Anderson R, Groundwater PW, Lough JW, et al Indolyl-3-acryloylglycine (IAG) is a putative diagnostic urinary marker for autism spectrum disorders. Med Sci Monit. 2003; 9(10): CR422–425. 14523330

[42] Bogen KT, Benson JM, Yost GS, Morris JB, Dahl AR, Clewell AJ, et al Naphthalene metabolism in relation to target tissue anatomy, physiology, cytotoxicity and tumorigenic mechanism of action. Regul Toxicol Pharmacol. 2014 doi: 10.1016/j.yrtph.2007.10.018 10.1016/j.yrtph.2007.10.018 PMC4030291 18191315

[43] Saeed M, Higginbotham S, Gaikwad N, Chakravarti D, Rogan E, Cavalieri E (2015). Depurinating naphthalene–DNA adducts in mouse skin related to cancer initiation. Free Radic Biol Med.2015 doi: 10.1016/j.freeradbiomed.2009.07.020 10.1016/j.freeradbiomed.2009.07.020 PMC4424927 19619639

